# RANDOMIZED CONTROLLED TRIAL OF LAY-DELIVERED INTERVENTION FOR DEPRESSED OLDER ADULTS

**DOI:** 10.1093/geroni/igad104.1342

**Published:** 2023-12-21

**Authors:** Amber Gum, Kyaien Conner, Nicole Crawford, Patrick Raue, Jo Anne Sirey

**Affiliations:** University of South Florida, Tampa, Florida, United States; University of Pittsburgh , Pittsburgh, Pennsylvania, United States; The University of South Florida, Tampa, Florida, United States; University of Washington, Seattle, Washington, United States; Weill Cornell Medicine, Ithaca, New York, United States

## Abstract

The first speaker will describe the background, aims, methods, and progress of an ongoing NIMH collaborative R01, three-site RCT. Across three sites (University of Washington, Cornell Weill Medicine, and University of South Florida), 288 depressed older adults will be enrolled, randomized to receive 9 sessions of BA from clinicians (e.g., social worker or mental health counselor) or 9 sessions of DMFB from a trained older adult volunteer coach, and assessed on primary outcomes (Behavioral Activation for Depression Scale [BADS], Hamilton Rating Scale for Depression [Ham-D]) and other outcomes at baseline, 3-, 6-, 9-, 24-, and 36-weeks. Clinician BA training and certification involves two 2-hour training workshops including didactics, demonstration, and role play; followed by delivering the intervention with one “certification” client reviewed and approved by external raters. Coach DMFB training and certification follow a similar process, except that training is longer (4 2-hour workshops) with more didactics, demonstrations, and role plays. After training, clinicians and coaches participate in regular weekly supervision, and all client sessions are audio-recorded, with at least one session per client randomly selected for independent review. This trial involves close collaboration with representative of a county lead aging service agency, numerous senior centers and organizations, and an Area Agency on Aging (AAA) for recruitment, outreach events, engagement of clinicians and volunteer coaches, and study dissemination. Across sites, 24 clinicians and 23 volunteers have been trained and certified, and 124 participants have been enrolled (as of February 15, 2023).

